# Physical origins of current and temperature controlled negative differential resistances in NbO_2_

**DOI:** 10.1038/s41467-017-00773-4

**Published:** 2017-09-22

**Authors:** Suhas Kumar, Ziwen Wang, Noraica Davila, Niru Kumari, Kate J. Norris, Xiaopeng Huang, John Paul Strachan, David Vine, A.L. David Kilcoyne, Yoshio Nishi, R. Stanley Williams

**Affiliations:** 1 Hewlett Packard Labs, 1501 Page Mill Road, Palo Alto, CA 94304 USA; 2 Stanford University, 350 Serra Mall, Stanford, CA 94305 USA; 3 Lawrence Berkeley National Laboratory, 1 Cyclotron Road, Berkeley, CA 94720 USA

## Abstract

Negative differential resistance behavior in oxide memristors, especially those using NbO_2_, is gaining renewed interest because of its potential utility in neuromorphic computing. However, there has been a decade-long controversy over whether the negative differential resistance is caused by a relatively low-temperature non-linear transport mechanism or a high-temperature Mott transition. Resolving this issue will enable consistent and robust predictive modeling of this phenomenon for different applications. Here we examine NbO_2_ memristors that exhibit both a current-controlled and a temperature-controlled negative differential resistance. Through thermal and chemical spectromicroscopy and numerical simulations, we confirm that the former is caused by a ~400 K non-linear-transport-driven instability and the latter is caused by the ~1000 K Mott metal-insulator transition, for which the thermal conductance counter-intuitively decreases in the metallic state relative to the insulating state.

## Abstract (web summary)

The development of future computation devices will be aided by a better understanding of the physics underlying material behaviors. Using thermoreflectance and spatially resolved X-ray microscopy, Kumar *et al*. elucidate the origin of two types of negative differential resistance in NbO_2_ memristors.

## Introduction

Negative differential resistance (NDR) in NbO_2_ is a manifestation of local activity that underlies threshold switching, generation of an action potential (in a neuristor), self-oscillations, and chaotic behavior, which are all being intensely researched for potential applications in neuromorphic or non-Boolean computing^1–9^. However, for several decades, there has been a controversy on whether the NDR is caused by a low-temperature (usually <500 K) non-linear-transport-driven thermal instability^4, 10–12^ or a high-temperature Mott metal-insulator transition (MIT)^1, 5, 13–17^. This issue is crucial for development of compact predictive models to study behaviors of larger computational systems constructed with NDR elements. Although accurate modeling of the low-current behavior consisting of the current-controlled NDR has been achieved recently^1, 4, 5, 11, 12^, a direct physicochemical measurement of the material temperature and localization effects during different NDR events have not been reported.

Here we examine nano and microscale NbO_2_ memristors that exhibit both a current-controlled and a less frequently observed temperature-controlled NDR. Through in operando thermoreflectance, in operando synchrotron X-ray transmission spectromicroscopy, transmission electron microscopy, and numerical simulations, we confirm that the current-controlled NDR is caused by a ~400 K non-linear Poole–Frenkel-transport-driven instability and the temperature-controlled NDR is caused by the ~1000 K Mott MIT. We highlight that temperature is the state variable for both types of NDRs, whereas they are driven by completely different physical mechanisms and occur at very different temperatures. We also show that the temperature and current density are spatially uniform over the memristor area through both NDRs when using a current source, whereas there is localized conduction channel/filament-formation when the memristor is biased in an unstable regime with a voltage source, recalling a long-standing and debated hypothesis^18, 19^.

## Results

### Fabrication and transmission electron microscopy

**Fig. 1:**
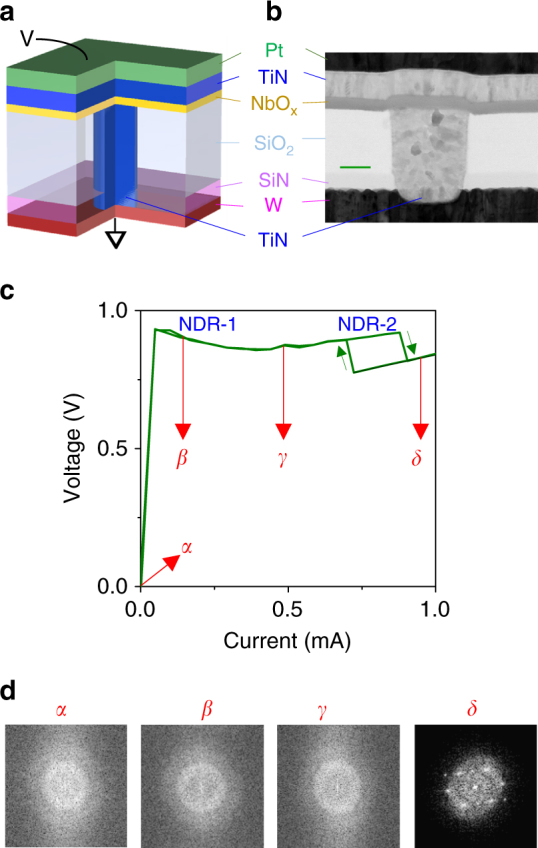
Memristor structure, electrical behavior, and crystallization upon operation. **a** Sectional schematic of the device. **b** Cross-sectional transmission electron micrograph of a device identical to the one used here. *Scale bar* is 25 nm. **c** A typical current–voltage curve obtained by sweeping current. Two regions containing negative differential resistance (NDR) behaviors, namely NDR-1 and NDR-2 are marked. *α–δ* are current levels representative of: an unoperated device (*α*), onset of NDR-1 (*β*), between NDR-1 and NDR-2 (*γ*), and beyond NDR-2 (*δ*). Electron diffraction patterns obtained using a transmission electron microscope within the active NbO_2_ layer on four different but nominally identical devices after operating them to different current levels (relative to NDR behaviors). Diffraction pattern at *δ* corresponds to a tetragonal [001] crystal projection, while the others were amorphous (as-grown)

We fabricated nanometer-scale NbO_2_ devices using a sub-100 nm diameter metallic TiN plug to contact a blanket thin film of amorphous NbO_2_ (Fig. 1a, b), the construction of which is described elsewhere (“Methods” section and Supplementary Figs. 2–4)^11^. The repeatable current–voltage curve (Fig. 1c) obtained by sweeping the applied current exhibits a region of current-controlled NDR (“NDR-1”), wherein the curve is single-valued for any value of current, and has been routinely observed in NbO_2_ before^1, 4, 5, 16^. However, at higher currents, this was followed by a rectangular hysteretic region consisting of a pair of sharp NDRs (“NDR-2”), which is neither current-controlled nor voltage-controlled. Current–voltage curves similar to the rectangular hysteresis have been observed in VO_2_ before and attributed to a Joule-heating-driven Mott MIT^20^. By studying the electron diffraction patterns of the initially amorphous active NbO_2_ layer across multiple devices, we observed irreversible crystallization only in devices that had been subject to current levels beyond those required to trigger NDR-2 (Fig. 1d). Since crystallization occurs at temperatures^21, 22^ in the range 800–1100 K, this observation suggests that NDR-2 is related to the MIT^23, 24^ (*T*_MIT_ is in the range of 1000–1100 K). Thus, NDR-1 was observed in both amorphous and crystalline NbO_2_, while NDR-2 was observed only in crystalline NbO_2_ (Supplementary Fig. 10).

### Temperature mapping using thermoreflectance

**Fig. 2:**
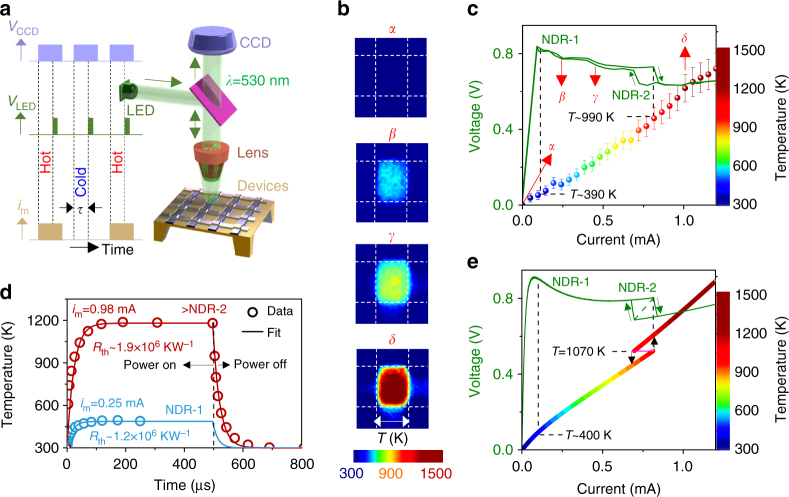
In operando thermoreflectance. **a** Schematic of the experimental setup. Synchronous operational pulse signals to the charge coupled device (CCD) full-field camera (*V*_CCD_), light-emitting diode (LED) (*V*_LED_), and the crosspoint devices (*i*_m_) are shown. “Hot” and “cold” refer to durations in which there is a current applied and not applied to the device, respectively. *τ* is the delay-time between the pump (application of *i*_m_) and probe (*V*_LED_). **b** Steady-state temperature maps of a single device at multiple constant current levels, with color-scale shown in **c**. *Scale bar* is 2 µm. **c** Experimental current–voltage curve of the device under measurement (using an increasing current sweep) and the corresponding average temperature within the crosspoint area at different current levels. Error bars represent the uncertainty in the measurement arising from calibration, noise, etc., (Supplementary Note 1). **d** Dynamic behavior of the average temperature within the crosspoint area of the same device upon application of a constant current (at time equal to 0) and upon withdrawal of the current (at time equal to 500 µs). **e** Simulated current–voltage curve and temperature of the device model (using increasing and decreasing current sweeps). *Black arrows* indicate hysteresis and temperature jumps predicted by the model. *Pink solid line*-segment indicates an abrupt jump in current that would occur upon a parametric temperature sweep. *Green dashed-line* segment is an abrupt jump in the current–voltage curve upon a parametric temperature sweep. *Black dashed lines* in **c**, **e** are used to indicate the temperature at the onset of the two regions of negative differential resistances (NDRs), namely, NDR-1 and NDR-2

In order to directly map the NbO_2_ temperature throughout the range of current–voltage operation, we employed in operando synchronous time-multiplexed pump-probe thermoreflectance with a spatial resolution of 290 nm (using incident light of wavelength 530 nm, Fig. 2a)^25^. While the nanometer-scale devices described above were suitable for transmission electron microscopy measurements, we required different structures for the thermoreflectance and synchrotron X-ray measurements. For these, we fabricated 2.0 × 2.5 μm crosspoint devices on 150-nm-thick silicon nitride membranes suspended over holes etched into a silicon substrate, with a material stack consisting of Pt (15 nm) (bottom electrode)/NbO_2_ (15 nm)/TiN (10 nm) (top contact)/Pt (15 nm) (top electrode). These devices exhibited similar quasi-static electronic behavior to the nanometer-scale devices examined previously when driven with a current source^26^, but there was only a single pinched hysteresis loop that encompassed both NDR-1 and NDR-2 when the device was driven with a voltage source (Supplementary Fig. 1). The thermal isolation of the structure resulted in insignificant temperature gradients normal to the membrane/device surface during the Joule heating due to the electrical operation of the devices, thereby enabling the thermoreflectance measurements on the device stack (of thickness <50 nm) to accurately represent the temperature of the active NbO_2_ layer^27^ (Supplementary Notes 1 and 2, Supplementary Figs. 6 and 7). The temperature maps (Fig. 2b) and the corresponding average temperatures within the crosspoint area (Fig. 2c) at different current levels (obtained using a current source) reveal that the temperature at the onset of NDR-1 is ~400 K, and that around NDR-2 is ~1000 K, which coincides with the MIT^1, 5, 13–17^. The temperature distribution within most of the crosspoint area (Fig. 2b) was spatially uniform across the entire current range, which indicates that the current density is also uniform. To estimate the thermal resistance of the device structure, we measured the temporal temperature evolution (rise and decay) within the crosspoint after application and withdrawal of electrical power (Fig. 2d). We then fitted the data to the dynamical equation for the state variable, which is the temperature (*T*), i.e., Newton’s law of cooling (Eq. 1) expressed in terms of the memristor current (*i*_m_), voltage (*v*_m_), and lumped thermal properties^11^,

1 $$\frac{{{\rm{d}}T}}{{{\rm{d}}t}} = \frac{{{i_{\rm{m}}}{v_{\rm{m}}}}}{{{C_{{\rm{th}}}}}} - \frac{{T - {T_{{\rm{amb}}}}}}{{{C_{{\rm{th}}}}{R_{{\rm{th}}}}\left( T \right)}},$$

where *T*_amb_ is the ambient temperature of 300 K; *C*_th_ is the thermal capacitance; and *R*_th_ is the effective thermal resistance. We determined that *R*_th_ for a steady-state temperature of ~490 K was 1.4 × 10^6^ ± 0.2 KW^−1^ (*T* < *T*_MIT_, current levels within NDR-1) and that for a steady-state temperature of >1100 K (*T* > *T*_MIT_, current levels above NDR-2) was 1.9 × 10^6^ ± 0.3 KW^−1^ (while we used the same *C*_th_ of 1.25 × 10^−11^ WsK^−1^ in both cases). While these estimates are obtained by crude simplifications of the values and functional forms of *R*_th_ and *C*_th_, they nonetheless agreed well with experimental data and provide a good starting point for further detailed modeling.

### Numerical modeling

To model the experimentally observed quasi-static current–voltage behavior, we adapt and extend a recently proposed modified three-dimensional Poole–Frenkel conduction mechanism with temperature as the state variable, represented by Eq. 2 ^4, 11^.

2 $${{i_{\rm{m}}} = \left[ {{\sigma _0}{e^{ - \frac{{{E_{\rm{a}}}}}{{{k_{\rm{B}}}T}}}}A\left\{ {{{\left( {\frac{{{k_{\rm{B}}}T}}{{\omega  }}} \right)}^2}\left( {1 + \left( {\frac{{\omega \sqrt {{v_{\rm{m}}}/d} }}{{{k_{\rm{B}}}T}} - 1} \right){{\rm{e}}^{\frac{{\omega \sqrt {{v_{\rm{m}}}/d} }}{{{k_{\rm{B}}}T}}}}} \right) + \frac{1}{{2d}}} \right\}} \right]{v_{\rm{m}}}},$$

where *A* is the lateral device area; *k*_B_ is the Boltzmann constant (in eV), *d* is the thickness of NbO_2_, and *σ*_0_, *E*_a_, and *ω* are material property constants described elsewhere^11^. To account for the MIT, following the results from the dynamic temperature measurements, we introduced an abrupt change in *R*_th_: 1.4 × 10^6^ (for *T* < *T*_MIT_) and 1.9 × 10^6^ (for *T* ≥ *T*_MIT_), where *T*_MIT_ = 1070 K. The quasi-static behavior described by Eqs. 1 and 2 (with $\frac{{{\rm{d}}T}}{{{\rm{d}}t}} = 0$) is in good agreement with experimentally measured data, especially in the reproduction of NDR-1 and NDR-2 (Fig. 2e). Significant increases in the complexity of the model are required to yield only moderate improvements in agreement with the experimental data, so we opt for simplicity here. The simulated temperatures of NbO_2_ across the applied current range are also in good agreement with the experimental measurements (Fig. 2c, e). The predicted hysteretic jumps during current-source operation across NDR-2 (*black* arrows) are too small to be observed experimentally, given the measurement uncertainty. They would change to a single-valued function of temperature (*pink solid line*) upon a parametric *T* sweep as displayed in Fig. 2e. Thus, NDR-2 is a temperature-controlled instability that manifests as a rectangular hysteresis during current-source operation. This behavior is similar to the Chua Corsage current–voltage characteristic^28^. Further, the counter-intuitive increase in *R*_th_ in the metallic state (*T* > *T*_MIT_) relative to the insulating/semiconducting state (*T* < *T*_MIT_), which is consistent for both temperature and electrical measurements (Supplementary Note 3, Supplementary Figs. 8 and 11), is opposite to that expected from the Wiedemann–Franz law. A recent report also described such anomalous behavior of *R*_th_ across the Mott transition in VO_2_ and provided plausible explanations that may cover the behavior observed here as well, although a comprehensive theory remains unexplored^29^.

### Synchrotron X-ray spectromicroscopy

**Fig. 3:**
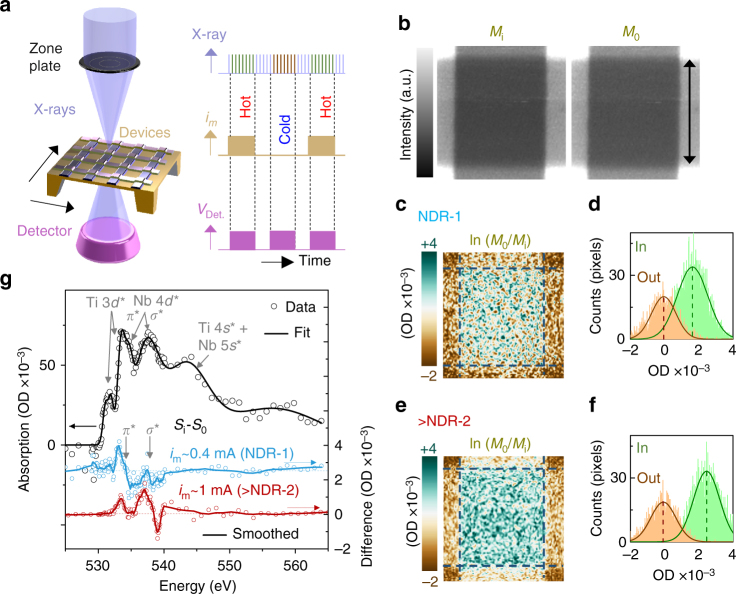
In operando synchrotron X-ray spectromicroscopy. **a** Schematic of the experimental setup. Synchronous operational pulse signals to the detector and the devices are shown, along with the incoming asynchronous X-ray pulses. **b** X-ray transmission maps of the crosspoint area of a fresh device identical to the one used in Fig. 2, with current (~0.4 mA, sufficient to cause NDR-1) (*M*_i_) and with no current (*M*_0_) obtained using 533.2 eV X-rays. *Scale bar* is 2 µm. **c** Logarithmic ratio of the maps in **b**. Intensity in optical density, OD. **d** Histograms of the data inside and outside the crosspoint area in **c**. **e** Logarithmic ratio of maps similar to those in (**b**) obtained with a current sufficient to exceed NDR-2 (~1 mA) and, (**f**) its corresponding histograms, similar to those in (**d**). **g** X-ray absorption spectra of the material within the crosspoint, along with the component bands marked. The spectral differences between the material with current and that with no current are also shown for two different currents, as marked (arbitrarily offset, *horizontal dashed lines* indicate zero difference for respective colors)

To further analyze the uniformity and the chemical nature of the NbO_2_ material changes in operando, we employed synchronous time-multiplexed scanning transmission X-ray microscopy (STXM) (Fig. 3a, Supplementary Note 4, Supplementary Fig. 12) using synchrotron radiation, which has been described elsewhere^30, 31^. This technique provided a spatial resolution of <30 nm and spectral resolution of 70 meV using X-ray energies tuned to the O K-edge, and the synchronous measurements enabled the detection of very low signal differences^26, 30^. For these experiments, we were able to use devices that were identical to the ones used for thermoreflectance, wherein the suspended thin silicon nitride membrane enabled transmitted X-ray detection. The X-ray maps of the crosspoint area (Fig. 3b) without a current (*M*_0_) and that with a current sufficient to cause NDR-1 (*M*_i_) do not show any noticeable differences between each other by eye. However, the logarithmic ratio of the maps (representing the optical density, OD) displays a small but detectable signal that appears to be uniform over the crosspoint area (Fig. 3c) and upshifted to higher values with respect to the material outside, revealing a chemical or electronic response to the flow of current near NDR-1. Both OD distributions were essentially Gaussian with means shifted by slightly <2 × 10^−3^ and essentially identical standard deviations (*S* ≈ 0.73 × 10^−3^), confirming that the changes were uniform. The reported data were averaged over several thousand measurement cycles, so the shift in the means is statistically significant^30^. A current sufficient to exceed NDR-2 caused a larger uniform shift of the OD to higher values (Fig. 3e, f, Supplementary Fig. 5, Supplementary Note 6). The O K-edge spectral difference between the material within the crosspoint with no current and that with a current sufficient to cause NDR-1 (Fig. 3g) displays a prominent feature at the rising edge of the π* band of NbO_2_^32, 33^, consistent with a downshifting of the lowest conduction band (“Methods” section). While the physical origin of this feature is uncertain, it could be caused by a lattice expansion of the NbO_2_ due to Joule heating at NDR-1^4, 11^. Also, the less pronounced signal in the difference spectrum corresponding to the Ti–O bond energies likely arise from a non-linear conduction mechanism in the TiO formed at the interface of TiN and NbO_2_ (Supplementary Figs. 2 and 3)^34^. Beyond NDR-2, a similarly obtained spectral difference displays prominent features consistent with downshifting of both the π* and σ* bands, in agreement with theoretical predictions of an increased conductivity at the MIT and an accompanying Peierls distortion in the crystal structure^24^. The Mott + Peierls transitions in NbO_2_ responsible for NDR-2 are similar to those in VO_2_ causing a behaviorally similar NDR^29, 35^ (discussed further in Supplementary Note 5 and Supplementary Fig. 13).

### Filament formation using a voltage source

**Fig. 4:**
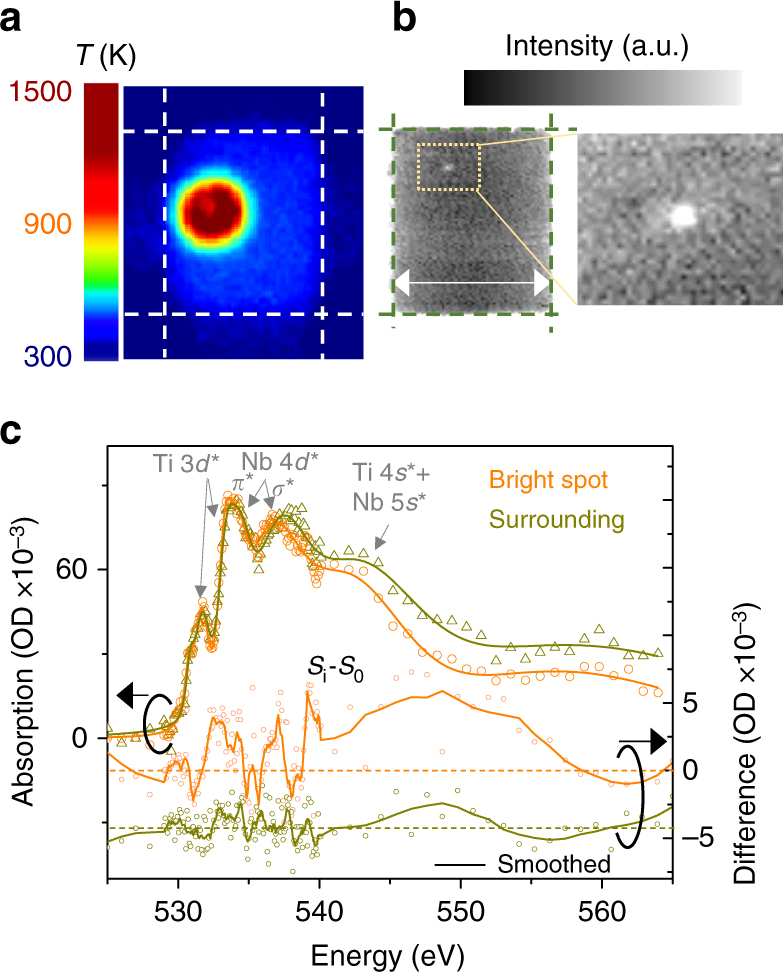
Operation with a voltage source. **a** Temperature map of a fresh crosspoint device identical to the ones explained previously held at an applied voltage of 1 V with *R*_S_ < *R*_NDR_). **b** X-ray transmission maps of a fresh crosspoint device identical to the one used in (**a**) that was previously subject to an applied voltage of 1 V (no bias was applied during acquisition of this data). *Scale bar* is 2 µm. A localized bright spot is shown in a magnified map. **c** X-ray absorption spectra of the “bright spot” and its “surrounding” region, color-coded to the legend. The spectral differences between the material with current and that with no current (*S*_i_–*S*_0_) are also shown for the two different regions (arbitrarily offset, *horizontal dashed lines* indicate zero difference for respective colors)

Following the observations of spatially uniform temperature and chemical changes during current-source measurements, we studied such responses when the devices were biased directly by a voltage source (with no external series resistance to limit the current; there was a series electrode resistance of about 300 Ω). Upon applying 1 V pulses (estimated current higher than 1.5 mA from the current–voltage sweeps shown in Supplementary Fig. 1, and thereby having accessed both the NDRs) to a crosspoint device using the thermoreflectance apparatus, we observed a localized hot-spot in the temperature map (Fig. 4a) that exhibited a temperature of more than 1200 K, while the rest of the crosspoint area had a relatively uniform temperature of ~400 K (corresponding to the onset of NDR-1). In a similar voltage-controlled experiment on a fresh but identical device probed using the time-multiplexed STXM technique, we observed the formation of a localized conduction channel, roughly 100 nm across, which persisted even after the voltage was removed, likely due to irreversible stoichiometry changes caused by the high temperatures (Fig. 4b, Supplementary Fig. 9). The post-O K-edge-absorption intensity within the channel was lower relative to its surroundings (Fig. 4c), revealing a lower O concentration. At the O K-edge, significant downshifting in energy of the component bands of NbO_2_ within the channel also indicated chemical reduction of the oxide^32, 33^. The spectral differences between the material with a 1 V and with zero bias also revealed a higher current density inside the channel^26^. The formation of a high current density “filament” or channel in a background of lower current density resulting from a symmetry-breaking instability in the NDR region was first proposed by Ridley based on entropy-production minimization arguments^18^, which were later questioned by Landauer^19^. Numerical simulations of NDR by Funck et al.^4^ also showed a high-current density channel within a lower-current density region, but this was enforced by the cylindrical symmetry of the model and the radial boundary conditions for heat flow. In our voltage source experiments, the NbO_2_ experienced irreversible material changes due to the high temperatures that were reached, so an unambiguous determination of the mechanism responsible for the channel formation is not possible from our data. Resolution of this interesting and important issue that is critical for robust modeling of NDR and other electrical instabilities will require further research.

## Discussion

Using spectromicroscopic characterization techniques to measure temperature and chemical spatial distributions, along with numerical simulations, we confirmed that the current-controlled NDR-1 in NbO_2_ is caused by a Joule-heating-driven thermal runaway in strongly nonlinear conduction at relatively low temperatures (~400 K) and further demonstrated that NDR-2 is a temperature-controlled MIT-driven hysteresis at high temperature (~1000 K). Both types of NDR within the same material are Joule-heating driven, and thus have *T* as a state variable, but they are caused by distinctly different physical mechanisms at very different temperatures. In addition, we showed that using a current source yields, a spatially uniform current density over the device area, while operating the devices with a voltage source causes the formation of a localized high current density channel, highlighting an outstanding problem on the subject of filament formation during electrical instabilities. These results definitively resolve the question of whether the NDR of NbO_2_ is caused by nonlinear temperature-dependent conduction or by a MIT—the answer is both mechanisms are distinct and present, which provides a rich set of nonlinear behaviors for potential exploitation.

## Methods

### Film growth

Si_3_N_4_ of 150 nm was grown using low-pressure chemical vapor deposition on double side-polished Si wafer with low p-doping. Holes were etched into the Si wafer to allow free suspension of Si_3_N_4_ membranes. Crosspoint cells were lithographically patterned onto these membranes. The Pt electrodes were evaporated from a Pt target. The layers of NbO_2_ and TiN were sputter-deposited.

### Spectral processing

Spectra were first normalized to background absorptions in the vacuum chamber, measured through a blank Si_3_N_4_ membrane (with no other material on it). The resulting spectra were corrected for a linear background in the pre-absorption-edge region. Peak fitting was done using the software Sigmaplot PeakFit. The pre-edge of the oxygen K-edge was aligned to zero, a linear background in the pre-edge region was subtracted, and the data was smoothed by 1% using Savitzky–Golay smoothing. The peaks were composed of a convolution of a Lorentzian component (to account for the broadening of spectral lines due to excited electron lifetime) and a Gaussian component (to account for the resolution of the spectrometer, mostly due to the beamline monochromator). For each spectrum, the component bands were allowed to vary in width, amplitude, and position to obtain the best fit. Smoothing of data in Figs. 3 and 4 was performed using Savitzky–Golay smoothing over 15 adjacent points.

### Data availability

The data that support the findings of this study are available from the corresponding author upon reasonable request.

## Electronic supplementary material

Supplementary Information
