# Supplementary material for: Physical origins of current and temperature controlled negative differential resistances in NbO2
Source: Nat Commun. 2017 Sep 22;8:658. doi: 10.1038/s41467-017-00773-4 (PMC5610340; doi:10.1038/s41467-017-00773-4)
Supplement: Supplementary file 1 — Supplementary Information [file 41467_2017_773_MOESM1_ESM.pdf]

Supplementary Information

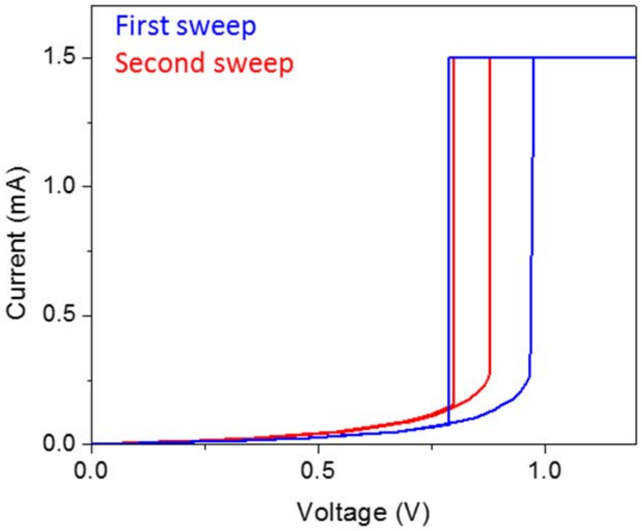

Supplementary Figure 1: Current-voltage behavior of NbO_2 obtained by voltage-sweep. A current limit of 1.5 mA was required while collecting this data to prevent the device from being destroyed by Joule heating. The resulting volatile threshold switching is shown. The first sweep required a slightly higher voltage compared to the subsequent repeatable sweeps and likely produced irreversible material changes. This data was obtained on a micrometer-sized crosspoint device. By comparing this result to the data obtained using a current source in Figs. 1 and 2, we can see that both NDR-1 and NDR-2 are contained within the voltage-sweep hysteresis loop.

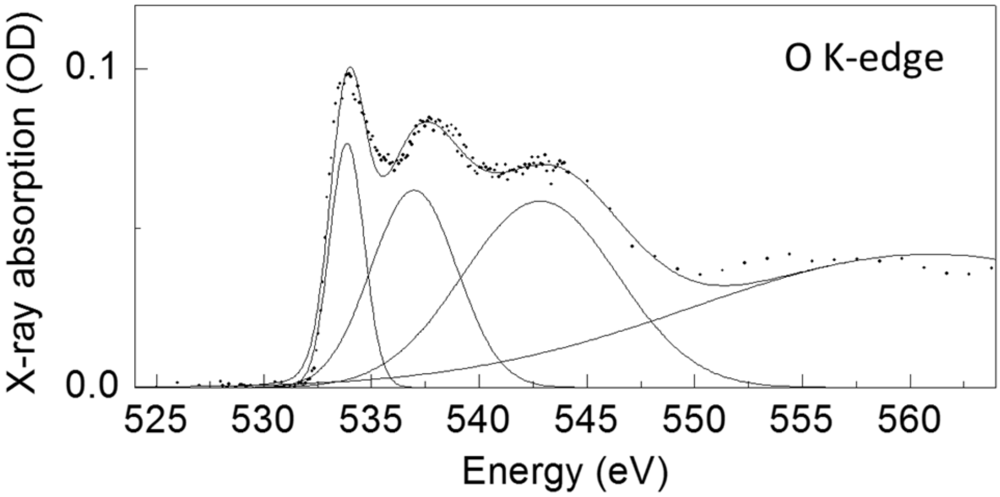

Supplementary Figure 2: As-grown NbO_2. O K-edge spectrum of as-grown NbO_2 (not in interface with any other oxides). The prominent bands of NbO_2 are clearly visible and identifiable.[1] This can be compared with the spectrum of the device stack (with niobium and titanium oxides) to identify the bands originating from Nb-O bonds.

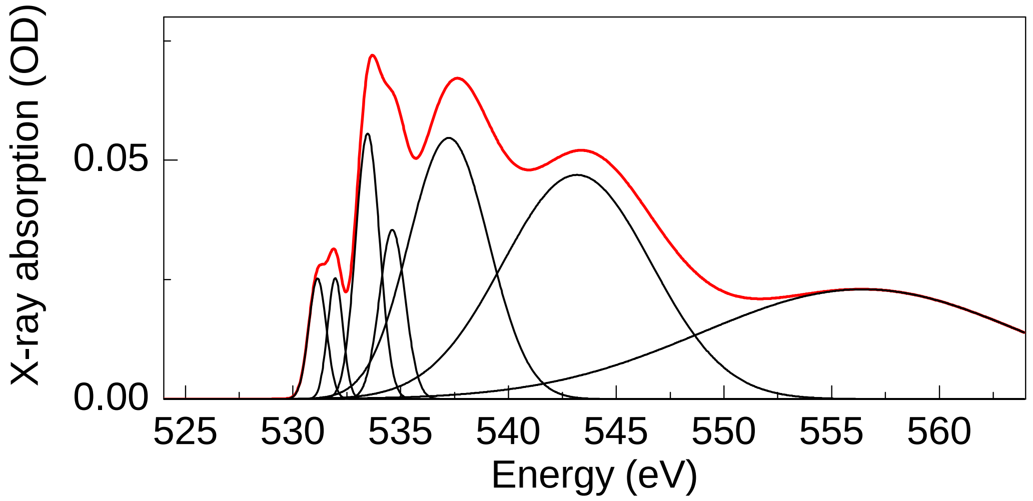

Supplementary Figure 3: Fit to the spectrum of the device stack. Fit to the O K-edge spectrum of the device stack (red) reproduced from Fig. 3, along with the component bands (black).

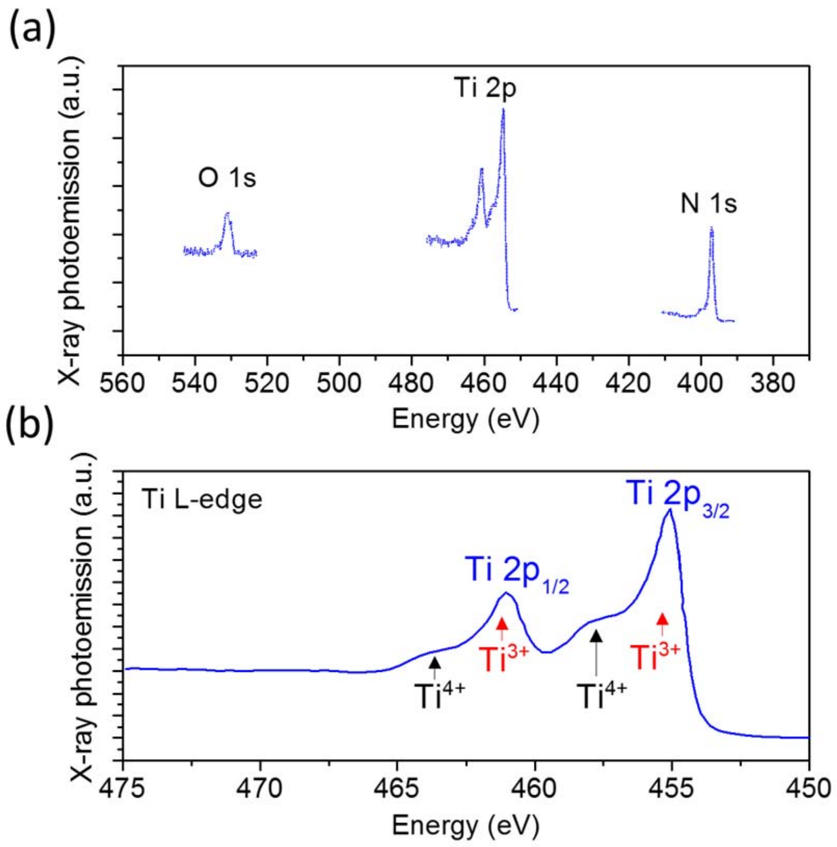

Supplementary Figure 4: As-grown TiN. (a) X-ray photoemission spectra of the O 1s, Ti 2p and N 1s peaks (as indicated) from an as-deposited film of TiN layer with no other oxide present. The oxygen contamination was found to be up to 19% (atomic percentage). (b) Magnified plot of the Ti L-edge spectrum from (a) showing that a majority of the Ti atoms were in the 3+ oxidation state, with a minority of them in the 4+ oxidation state. It is plausible that the power-driven sputtering of this film onto the underlying niobium oxide layers during fabrication of a cell stack drove some of the Ti ions into the niobium oxide layers, thereby causing further oxidation of Ti. This is likely the origin of the Ti-O signal observed in the x-ray spectra (Fig. 3).

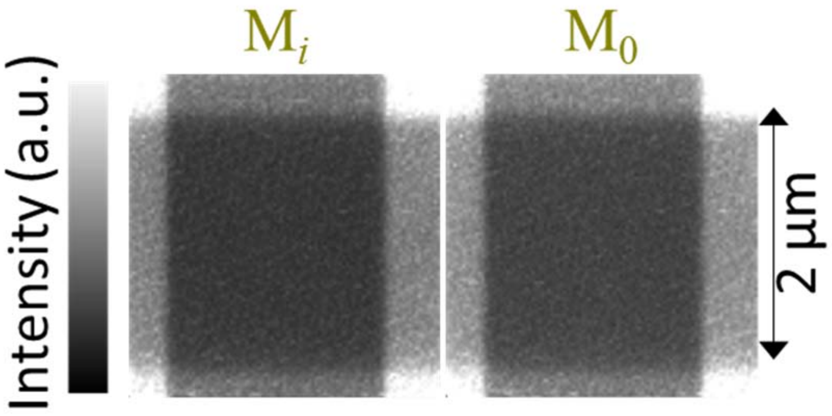

Supplementary Figure 5: Raw data of Fig. 3e. Component x-ray maps, the ratio of which is shown in Fig. 3e obtained using 533.2 eV x-rays.

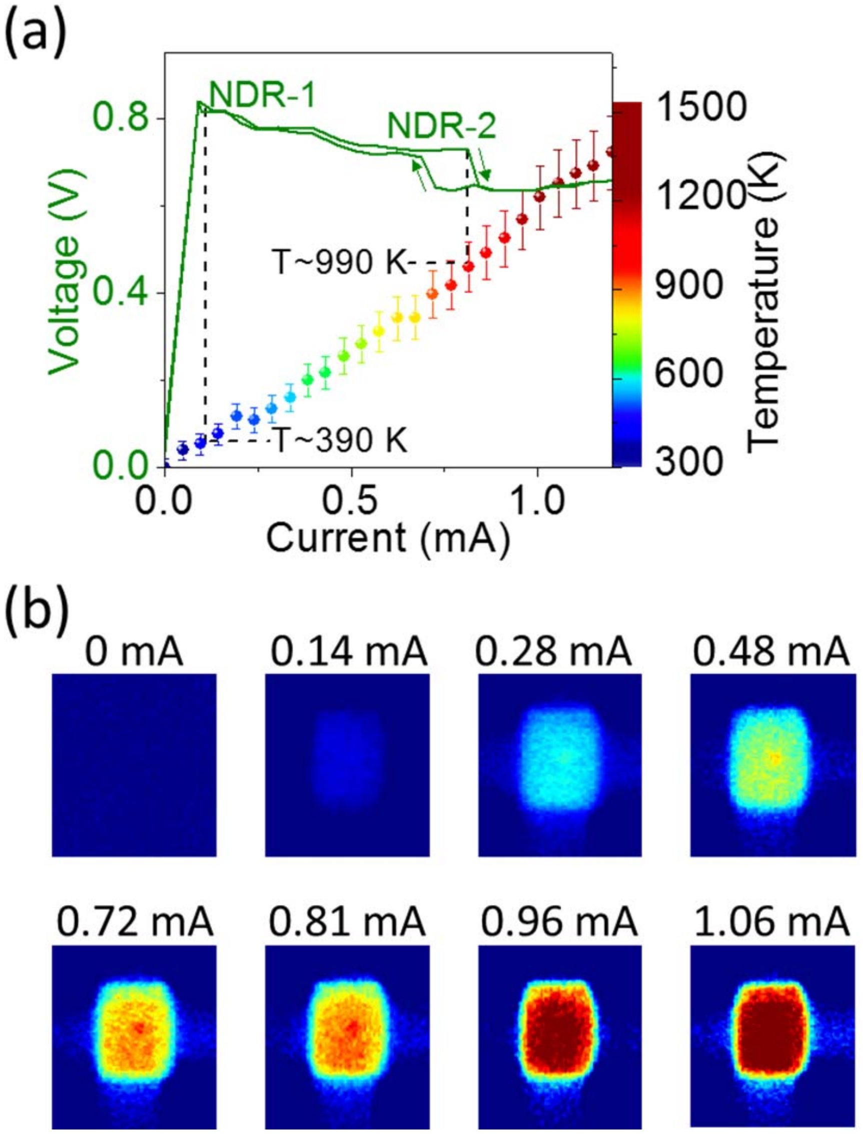

Supplementary Figure 6: Extended data of Fig. 2c. (a) Reproduction of Fig. 2c. (b) Extended data-set of temperature maps obtained at different currents corresponding to Fig. 2c. Color-scale is shown in (a)

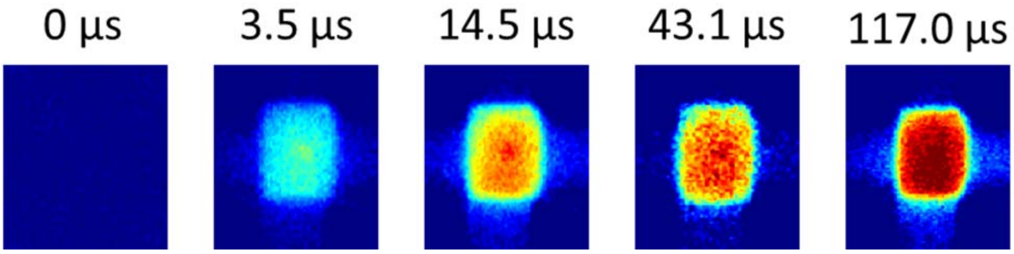

Supplementary Figure 7: Raw data corresponding to Figure 2d. Temperature maps corresponding to the temporal temperature evolution presented in Fig. 2d for an applied current of 0.98 mA. Color-scale is shown in Supplementary Fig. 6.

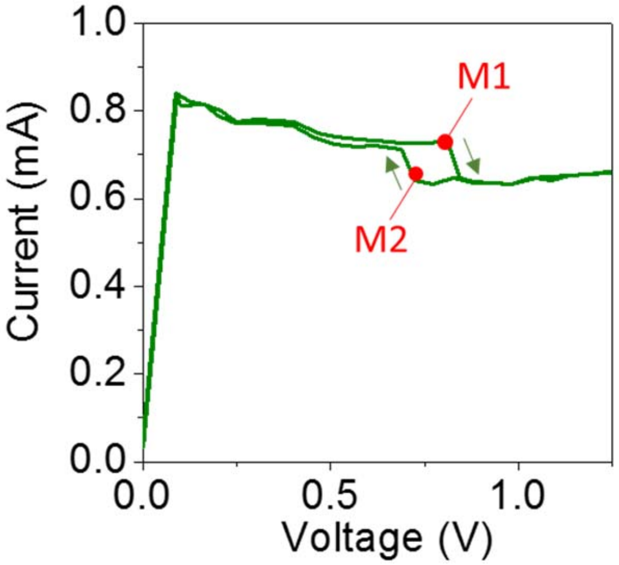

Supplementary Figure 8: Calculation of the ratio of change in R_{th} from the static current-voltage curve. Current-voltage curve of the device repeated from Fig. 2c. M1 and M2 refer to two MIT events during ramping-up and ramping-down of current, respectively.

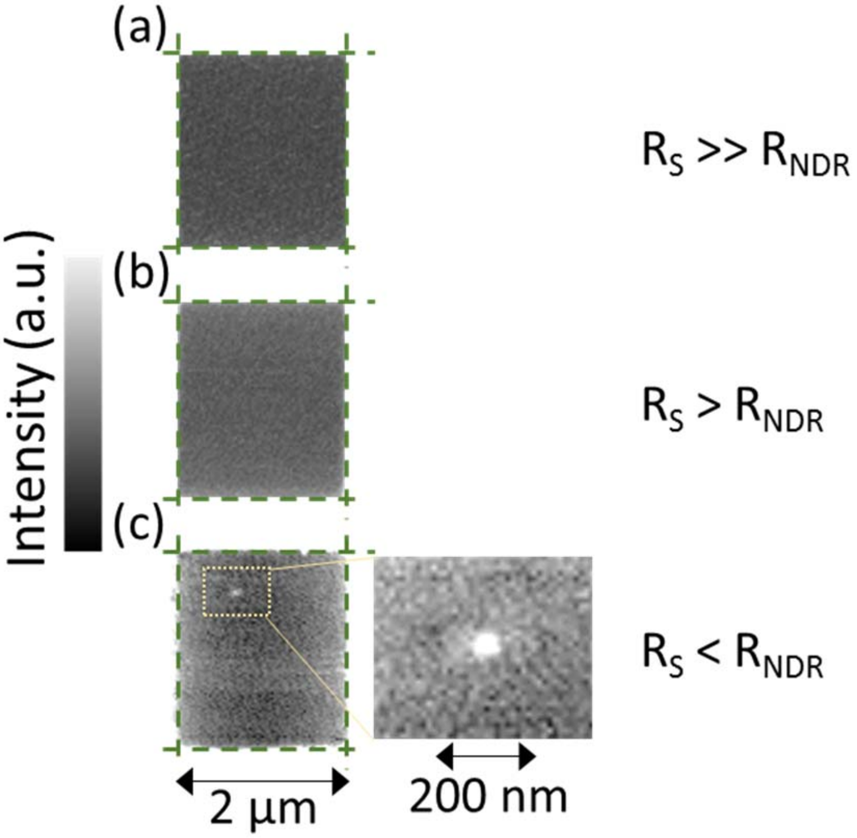

Supplementary Figure 9: Comparing uniformity of switching for different series resistances (R_S). (a) X-ray map of a device operated using current-sweep (i.e.: a voltage source with a very large series resistance (R_S)), which was much greater than the magnitude of NDR, R_{NDR} (200-400 Ω). (b) X-ray map of a device operated with voltage source and a series resistance of ~3 kΩ (R_S>R_{NDR}). X-ray map of a device operated with a voltage source and a series resistance (from the contact electrodes) of about 300 Ω (R_S<R_{NDR}). These maps show that as R_S is reduced below R_{NDR}, there is a localized conduction channel that forms and causes an irreversible change to the NbO_2.

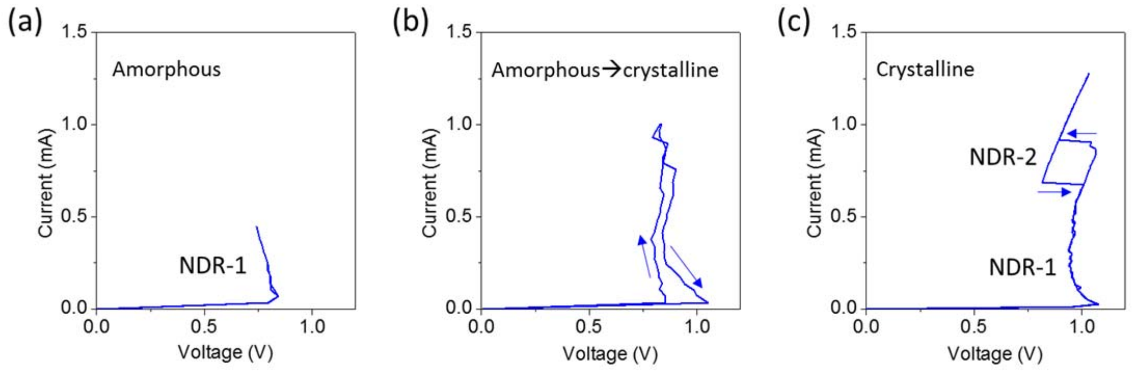

Supplementary Figure 10: NDR observed in amorphous and crystalline NbO_2. (a) Repeatable current-voltage curve of a device which has not been subjected to current above those required to cause NDR-2. These current levels also did not cause irreversible crystallization of the NbO_2 layer. Thus, the NDR-1 (current-controlled NDR) observed here represents the behavior of the amorphous NbO_2. (b) Current-voltage curve of the same device in (a) upon the first instance of subjecting it to current levels above those required for NDR-2. The sharp changes (that were irreproducible and irreversible) are most likely associated with the irreversible crystallization that was confirmed by electron microscopy. (c) Repeatable current-voltage plot of the same device after the behavior observed in (b). The NDR-1 and NDR-2 observed here represent the behavior of the crystalline NbO_2.

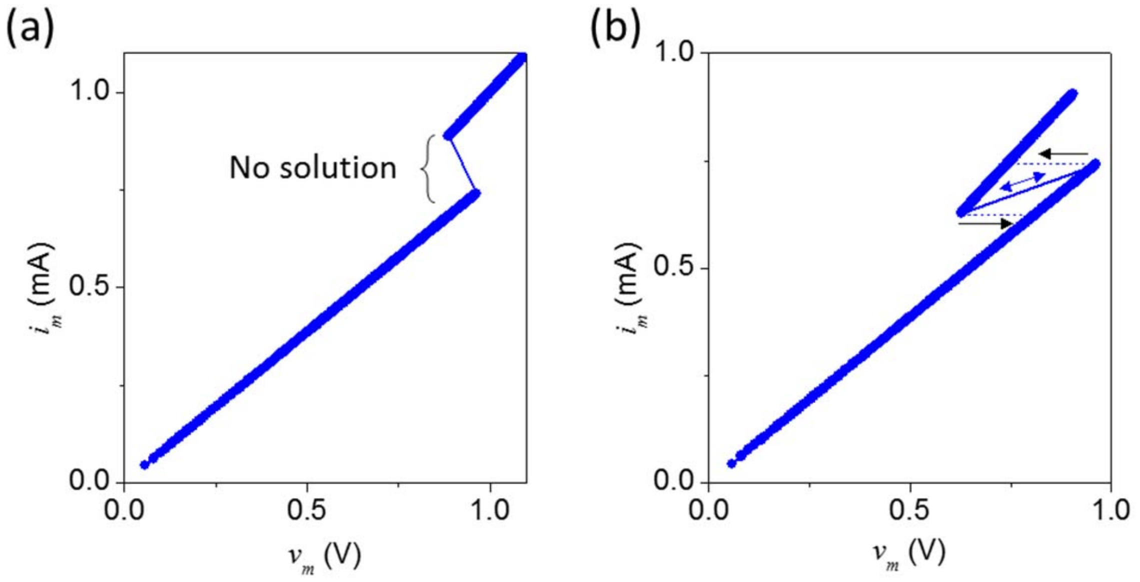

Supplementary Figure 11: A simple conductance change during the Mott transition cannot reproduce the box-shaped hysteresis behavior (NDR-2). (a) Simulation of a simple linear resistor of

value 1000 Ω (at low temperatures), which changes resistance value abruptly to 500 Ω at a critical temperature of 1070 K. This yields a current-voltage plot with a range of current values for which there is no solution of the resulting curve (solid blue line with no data-markers). This leaves the system unstable in this current range, leading the system to utilize another state parameter to achieve stability. This is often some material property (e.g.: R_{th}) or formation of localized conduction channels, wherein the cross-sectional area of the conduction channel becomes the state parameter. (b) An identical simulation as in (a), except that there is an increase in R_{th} at the critical temperature, as used in the rest of the manuscript. The solutions for a sweep of temperature across different values are shown with solid blue circles, with a solid blue line connecting them. Blue dashed lines are solutions to a current sweep, marked with black arrows. Thus, we establish the importance of identifying the material property change during a Joule-heating-driven Mott transition. Thus, a simple conductance change during the Mott transition cannot reproduce the box-shaped hysteresis behavior (NDR-2). This eliminates area of the conducting channel as a state parameter in the model. An accompanying abrupt increase in R_{th} in the metallic state is required.

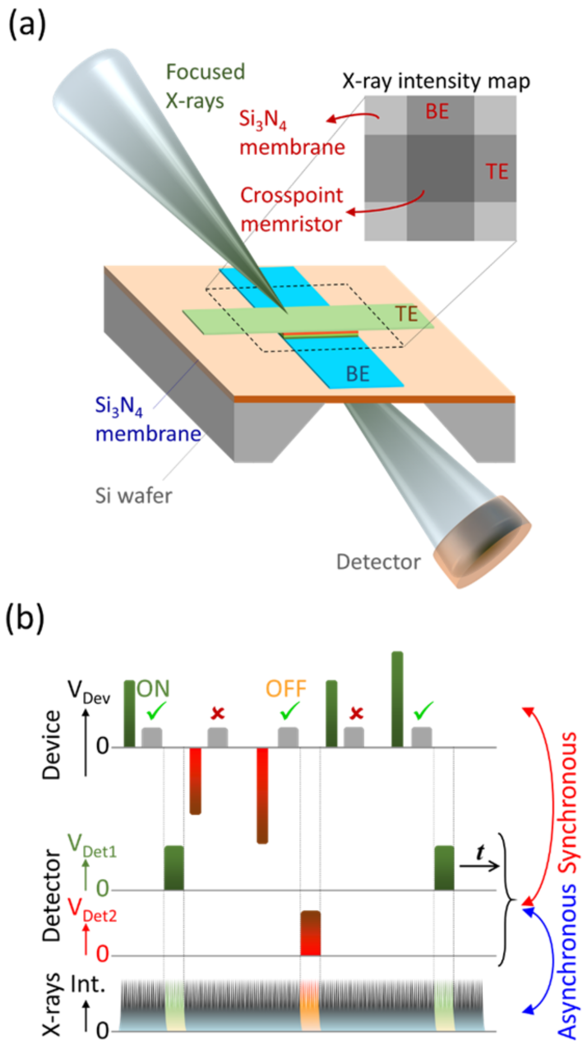

Supplementary Figure 12: The time-multiplexed high-signal x-ray system. (a) Schematic of the STXM measurement setup and the cell under measurement. Also shown is a schematic of the x-ray intensity map obtained by imaging the crosspoint region of cell structure, with the top electrode (TE) and the bottom electrode (BE) identified. (b) Schematic of the adaptive time-multiplexed technique applied to the STXM measurements. The device voltage (V_{Dev}) consisted of bipolar voltage pulses (green and orange) to continually switch the resistance of the cell, and subsequently read the resistance (grey pulses) to verify that a pre-determined threshold for the ON or OFF state of the device was reached ('✓'), while a failure ('✗') resulted in repeated attempts with larger voltage pulses. Detector voltages (V_{Det1} and V_{Det2}) were synchronously applied only upon verification of a successful resistance change to store signals from the ON and OFF states in two separate counters, obtained by measuring the asynchronous x-ray intensity (x-rays, int.) during detection.

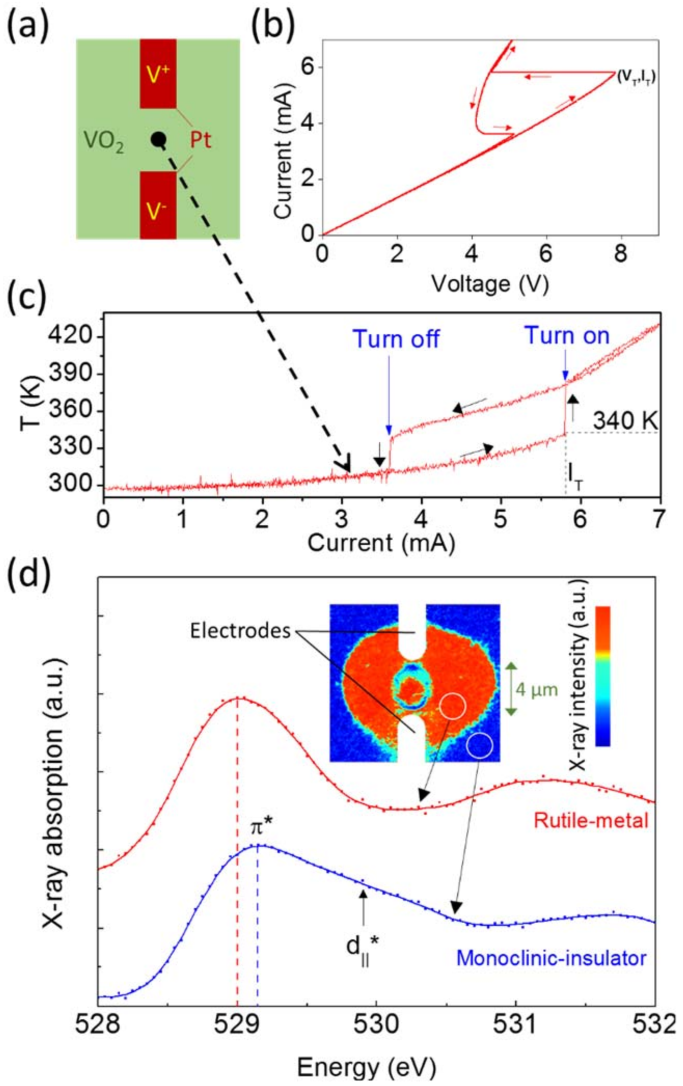

Supplementary Figure 13: Electrical, temperature and x-ray measurements on VO_2. (a) Device structure, with lateral Pt electrodes deposited over a blanket VO_2 film. (b) Current-voltage behavior obtained by sweeping current. It is noted that, unlike NbO_2, there is only NDR-2 that is observed but NDR-1 is not observed. (c) Local temperature measured between the two electrodes at different currents. 'Turn on' and 'Turn off' refer to the current levels at which the voltage undergoes abrupt changes in (b). (d) *In-operando* x-ray spectromicroscopy on a fresh device showing that the there was a crystal structure change (Peierls transition) accompanying a Mott transition (shifts in the lowest conduction bands) upon driving a current higher than that required for causing NDR-2.

Supplementary Note 1: Additional details on temperature and electrical measurements

Quasi-static current-voltage behavior was measured using an Agilent B1500 parameter analyzer and a Cascade probe station. The parameter analyzer was controlled through a General Purpose Interface Bus (GPIB) using software programs written in Igor. The voltages measured across the device were measured over 1-10 ms at every data-point, which was sufficiently long to allow for attainment of steady-state. Thermoreflectance measurements were performed using a Microsanj NT220 thermoreflectance analyzer, the details of which (including construction, operating principles, calibration procedures, etc.) are available elsewhere.[2] The typical error we expected from the measurements were ~5% (of measured temperature) dependent on measurement parameters including time-of integration, number of averaging cycles, noise, etc. To account for other sources of errors like drift in sample behavior, noise/distortions in the drift-correcting piezoelectric stages, etc., we report a total error of 10% in Fig. 2c. Thermoreflectance measurements involved repeated cycling of the devices through many cycles of high and low current levels (through time-multiplexing) using pulsed current signals. The wait-time ('τ' in Fig. 2a) was set to at least 1 ms for acquisition of steady-state data reported in Fig. 2. Attainment of steady-state within 1 ms was verified by collecting dynamic temperature data by varying τ (Fig. 2d). In all the experimental measurements, a study of NDR-2 was performed using current levels sufficient to exceed NDR-2, because it is impossible to achieve a stable access of NDR-2 using any combination of electrical parameters, since NDR-2 is a manifestation of a temperature-controlled instability.

Supplementary Note 2: Temperature gradients within the device structure

Since the temperature maps show a fairly uniform temperature distribution within the crosspoint area and no significant temperature gradients outside the crosspoint area, it is fair to assume that heat transfer in the lateral direction is negligible with regard to the measurements. Along the direction perpendicular to the membrane/device surface, we consider the following heat transfer equation:

$$(T_{NbO} - T_S)\frac{k_f}{t_f} = h_{air}(T_S - T_{amb}) + \sigma_s(T_S^4 - T_{amb}^4) \ , \qquad \text{(Supplementary Equation 1)}$$

where T_{NbO} is the temperature of the niobium oxide layer; T_S is the temperature at the surface of the material stack; k_f is the effective thermal conductivity of the layers contacting the niobium oxide layer, with a total thickness of t_f; h_{air} is the heat transfer coefficient of air that also accounts for convection (100 $Wm^{-2}K^{-1}$); σ_s is the Stefan's constant (5.6×10^{-8} $Wm^{-2}K^{-4}$); the ambient temperature of the bath, T_{amb}, was set to 300 K. Supplementary Equation 1 accounts for heat transfer through the solid layers in contact with

the niobium oxide, through air (including convective cooling) and by radiation (assuming a perfect black-body). We used extreme-case values for k_f of 1 $Wm^{-1}K^{-1}$, and for t_f of 200 nm (considering the 150 nm of freely suspended silicon nitride membrane beneath the device and the other layers in the stack). By solving Supplementary Equation 1, we found that the difference between T_{NbO} and T_S for all values of T_{NbO} between 300.1 K and 1500 K was <0.1 K. This shows that the temperature gradient across the thickness of the silicon nitride membrane (150 nm) was sufficiently insignificant, thereby allowing us to ignore such temperature gradients across the thickness of the electrodes (<20 nm).

Supplementary Note 3: Estimating the R_{th} jump from the electrical data

Using the static current-voltage sweep, it is possible to estimate the magnitude of the abrupt change in R_{th} upon MIT. Consider the MIT events M1 (insulator state (Ins) at the onset of insulator-to-metal transition) and M2 (metallic state (Met) at the onset of metal-to-insulator transition) occurring during increase and decrease of current, respectively, as marked in Supplementary Fig. 8. Assuming that the MIT occurs at a constant temperature,[3] we can represent T_{MIT} as:

$$\mathrm{T_{MIT}} = i_m^{\mathrm{Ins}} v_m^{\mathrm{Ins}} \mathrm{R_{th}^{Ins}} = i_m^{\mathrm{Met}} v_m^{\mathrm{Met}} \mathrm{R_{th}^{Met}} \qquad \text{(Supplementary Equation 2)}$$

$$\mathrm{R_{th}^{Ins}} / \mathrm{R_{th}^{Met}} = i_m^{\mathrm{Met}} v_m^{\mathrm{Met}} / i_m^{\mathrm{Ins}} v_m^{\mathrm{Ins}} \qquad \text{(Supplementary Equation 3)}$$

where the superscripts (Ins and Met) denote the quantities measured at the specified MIT events M1 or M2, respectively. Thus, it is possible to calculate the ratio of change in R_{th} from the currents and voltages measured at M1 and M2. From this, we estimated the ratio in Supplementary Equation 3 to be 0.77 from the current-voltage curve, while that obtained from direct temperature mapping was 0.74. This good agreement between the two estimates obtained from different experiments supports the larger conclusion of an increase in R_{th} in the metallic state of NbO_2.

Supplementary Note 4: *In-operando* x-ray characterization technique

One of the greatest challenges in physically understanding memristor operation has been the extremely localized, low signal, atomic-scale material changes associated with large changes in the cell resistance, the result of which is uncertainty and controversy on the details of the operating mechanisms.[4] Experiments are usually performed using destructive techniques like cross sectional electron microscopy, non-standard device construction, and amplifying the material changes by using stronger operating conditions.[5,6] To overcome these limitations, we developed an ultra-sensitive measurement technique to probe the electronic, structural, and chemical properties during regular operation of a cell.[4,7] In order to achieve the necessary spatial and spectral resolution, we employed a synchrotron-based scanning

transmission x-ray microscope (STXM) with spatial resolution of <30 nm and spectral resolution of ~70 meV.[8] Prototype devices for this experiment were fabricated atop 200 nm thick freely suspended Si_3N_4 membranes to enable x-ray transmission.[9] Further, in order to overcome the signal-limiting issues of spatial drift, background absorption changes, stochasticity in cell operation and drift in cell behavior, we constructed an *in-operando* time-multiplexed experimental setup (Supplementary Fig. 12). We incorporated an adaptive cell-switching technique that utilized feedback-enabled resistance switching together with a verification read, while synchronously gating the detector signal (from the asynchronous synchrotron x-ray pulses) into two different counters corresponding to physical measurements only when the target resistance states were successfully achieved. The result was an integration of signal at every spatial and spectral position over many verified switching events, which averages stochastic processes during cell switching. More importantly, the time-multiplexing provided a reduction in effects due to spatial drift of the sample and other background changes by over 5 orders of magnitude, detailed elsewhere.[4] The adaptive system also corrected for temporal drifts in cell switching behavior, which is another major source of distortion in such measurements.

Supplementary Note 5: Comparison of electrical, temperature and x-ray measurements to VO2

In Fig. 2c the abrupt jump in temperature predicted by the model in Fig. 2e is not observed, mainly because the size of the effect is within the error bars on the experimental data. Since NbO_2 undergoes a Mott + Peierls transition at ~1000 K, which is challenging to perform accurate thermal measurements on, we show here a similar effect in VO_2, a Mott insulator that undergoes a Mott + Peierls transition at ~340 K, which is easier to measure/calibrate. In Supplementary Fig. 13, we show a set of previously published data that reproduces the current-driven 'four-sided' hysteretic NDR-2 measured in VO_2 (Supplementary Fig. 13b), along with local temperature measurement (Supplementary Fig. 13c) and *in-operando* x-ray spectromicroscopy (Supplementary Fig. 13d).[3,9] The local temperature is 340 K at the threshold current, which causes the sharp NDR-2 and is the precise Mott transition temperature of VO_2. Having established that we are indeed studying the Mott transition-driven NDR of VO_2, we also see that the temperature undergoes abrupt hysteretic jumps accompanying the Mott transition and NDR-2, consistent with our MIT model. This also shows that there are abrupt jumps in R_{th} for VO_2. Further, using x-ray spectromicroscopy, we showed that there is a Peierls transition that accompanies NDR-2, the Mott transition and the abrupt change[9] in R_{th} of VO_2. This data details the crystal phases that are associated with the different R_{th}. Similar x-ray absorption data has been provided for NbO_2 as well (Fig. 3). However, VO_2 does not exhibit NDR-1 because the MIT occurs at a temperature lower than the nonlinear conductance thermal runaway regime of NbO_2. Another oxide, TiO_2, exhibits NDR-1 but not NDR-2,

because it does not have a Mott transition.[10] Thus, NbO_2 is seen to be unusual if not unique in its nonlinear electronic behavior.

Supplementary Note 6: Statistical analysis of the OD distributions

In Fig. 3, the mean of the OD inside the crosspoint area (M ≈ 1.62×10^{-3} in Fig. 3d and M ≈ 2.47×10^{-3} in Fig. 3f) contained a relatively negligible error, M_{err}, estimated from the sample standard deviation as M_{err} = $S/\sqrt{N}$ ≈ 1.622×10^{-7} for N>3500 representing many measurement cycles. The standard deviations (S ≈ 0.73×10^{-3}) were similar for all the distributions reported in Fig. 3 and was a function of the dwell-time of the focused x-rays within each pixel of the map.

Supplementary References

1 Soriano, L., Abbate, M., Fuggle, J. C., Jimenez, M. A., Sanz, J. M., Mythen, C. & Padmore, H. A. The O 1s x-ray absorption spectra of transition-metal oxides: The TiO2-ZrO2-HfO2 and V2O5-Nb2O5-Ta2O5 series. *Solid state communications* **87**, 699-703, (1993).

2 Farzaneh, M. *et al.* CCD-based thermoreflectance microscopy: principles and applications. *Journal of Physics D: Applied Physics* **42**, 143001, (2009).

3 Kumar, S., Pickett, M. D., Strachan, J. P., Gibson, G., Nishi, Y. & Williams, R. S. Local Temperature Redistribution and Structural Transition During Joule-Heating-Driven Conductance Switching in VO2. *Advanced Materials* **25**, 6128-6132, (2013).

4 Kumar, S., Graves, C. E., Strachan, J. P., Kilcoyne, A. L. D., Tyliszczak, T., Nishi, Y. & Williams, R. S. In-operando synchronous time-multiplexed O K-edge x-ray absorption spectromicroscopy of functioning tantalum oxide memristors. *Journal of Applied Physics* **118**, 034502, (2015).

5 Park, G.-S. *et al.* In situ observation of filamentary conducting channels in an asymmetric Ta2O5−x/TaO2−x bilayer structure. *Nat Commun* **4**, (2013).

6 Kumar, S., Graves, C. E., Strachan, J. P., Grafals, E. M., Kilcoyne, A. L. D., Tyliszczak, T., Weker, J. N., Nishi, Y. & Williams, R. S. Direct Observation of Localized Radial Oxygen Migration in Functioning Tantalum Oxide Memristors. *Advanced Materials* **28**, 2772-2776, (2016).

7 Kumar, S., Davila, N., Wang, Z., Huang, X., Strachan, J. P., Vine, D., Kilcoyne, A. D., Nishi, Y. & Williams, R. S. Spatially uniform resistance switching of low current, high endurance titanium-niobium-oxide memristors. *Nanoscale* **9**, 1793, (2016).

8 Kilcoyne, A. L. *et al.* in *J Synchrotron Radiation* **10,** 125-136 (2003).

9 Kumar, S., Strachan, J. P., Pickett, M. D., Bratkovsky, A., Nishi, Y. & Williams, R. S. Sequential Electronic and Structural Transitions in VO2 Observed Using X-ray Absorption Spectromicroscopy. *Advanced Materials* **26**, 7505-7509, (2014).

10 Alexandrov, A. S., Bratkovsky, A. M., Bridle, B., Savel'ev, S. E., Strukov, D. B. & Williams, R. S. Current-controlled negative differential resistance due to Joule heating in TiO2. *Applied Physics Letters* **99**, 202104, (2011).
